# From Cell States to Cell Fates: How Cell Proliferation and Neuronal Differentiation Are Coordinated During Embryonic Development

**DOI:** 10.3389/fnins.2021.781160

**Published:** 2022-01-03

**Authors:** Carla Belmonte-Mateos, Cristina Pujades

**Affiliations:** Department of Experimental and Health Sciences, Universitat Pompeu Fabra, Barcelona, Spain

**Keywords:** neural stem cells (NSC), neurogenesis, neuronal differentiation, morphogenesis, cell fate, cell state

## Abstract

The central nervous system (CNS) exhibits an extraordinary diversity of neurons, with the right cell types and proportions at the appropriate sites. Thus, to produce brains with specific size and cell composition, the rates of proliferation and differentiation must be tightly coordinated and balanced during development. Early on, proliferation dominates; later on, the growth rate almost ceases as more cells differentiate and exit the cell cycle. Generation of cell diversity and morphogenesis takes place concomitantly. In the vertebrate brain, this results in dramatic changes in the position of progenitor cells and their neuronal derivatives, whereas in the spinal cord morphogenetic changes are not so important because the structure mainly grows by increasing its volume. Morphogenesis is under control of specific genetic programs that coordinately unfold over time; however, little is known about how they operate and impact in the pools of progenitor cells in the CNS. Thus, the spatiotemporal coordination of these processes is fundamental for generating functional neuronal networks. Some key aims in developmental neurobiology are to determine how cell diversity arises from pluripotent progenitor cells, and how the progenitor potential changes upon time. In this review, we will share our view on how the advance of new technologies provides novel data that challenge some of the current hypothesis. We will cover some of the latest studies on cell lineage tracing and clonal analyses addressing the role of distinct progenitor cell division modes in balancing the rate of proliferation and differentiation during brain morphogenesis. We will discuss different hypothesis proposed to explain how progenitor cell diversity is generated and how they challenged prevailing concepts and raised new questions.

## A Short Historic Glance at Cell Fate

The making of an embryo entails the production of billions of specialized cells from a single pluripotent cell, the zygote, and their organization into tissues and organs. During embryonic development, stem cells must balance self-renewal, commitment to specific fates and differentiation to generate the wide diversity of cells in the correct numbers and proportions to construct functional organs. The embryo undergoes morphogenesis, which consists of specific tissue changes occurring orderly in time. This results in a multitude of tissue and organism shapes, which are controlled by fundamental processes involving cell mechanics. The high reproducibility of embryonic development argues that these events are tightly spatiotemporally regulated and that embryonic cells interpret specific information that organizes their behavior. Thus, to learn how to construct functional organs we need to elucidate the mechanisms that regulate how cell proliferation, specification and differentiation occur alongside morphogenesis. Or in other words, how gene regulatory networks (GRN) encode tissue shape.

These questions, such as how cells acquire their fate, have fascinated scientists for centuries. Experiments from the 1890s led to the emergence of the hypothesis that developmental mechanisms regulate the differentiation of different cell types occurring on a developmental landscape sculpted by genes (Waddington, 1957; for reviews covering this topic see Slack, 2002; Takahashi and Yamanaka, 2016; Collinet and Lecuit, 2021). This deterministic view considered that genes defined all developmental cell trajectories and gave rise to the *mosaic theory of development* in which the fate of each cell in an embryo was specified very early and followed fixed developmental trajectories. This implied a crucial role for cell-autonomous factors and that cells –once committed– could not change their fate. The publication of the stereotyped cell lineages trees of *Caenorhabditis elegans* in 1983 (Sulston et al., 1983), showing that the segregation of genetic determinants at each cellular division defined the different cell populations in the progeny, consolidated this view. The identification of *morphogens*, secreted molecules whose concentration conferred positional information within a field of cells, and the discovery of the *master genes*, which were able to drive the entire genetic cascade to form an organ, reinforced the idea of a genetic program controlling development. In spite of it, several observations from experimental embryologists in the early XX century suggested that development resulted from more than deterministic rules (Rogers and Schier, 2011). Experimental embryology manipulations mainly in amphibians indicated that during development cell–cell and cell–environment interactions led cells to adopt a particular fate in a not predetermined manner. The discovery, by Brown in hydra and Spemann and Mangold in amphibians, that groups of cells –inductors– could change the fate of neighboring competent cells challenged again the *mosaic theory*. Development could proceed by selection of a few viable dynamical cellular states, which resulted from local cell–cell interactions occurring within the embryo in a self-organized manner. This led to the idea that non–cell-autonomous factors were needed for cells to acquire different functions. This has been called the *regulative view of development* (for reviews see Robertis, 2006; Rogers and Schier, 2011). Now, we know that both deterministic and self-organization programs play important roles.

In this review, we will focus on how cell behaviors and neuronal fates are deployed during the development of the vertebrate Central Nervous System (CNS). Specifically, we will cover some examples of how neural progenitor cells transition through different proliferation modes to finally differentiate, and the implications for the overall growth and morphogenesis of the CNS. Due to the vast and unattainable literature, attention will be paid to some of the latest cell lineage and clonal studies, since they inform us about the role that **time** plays in the deployment of the different cell fates –a crucial factor not very much addressed up to now. We will focus on two of the paradigm models for tissue growth in the vertebrate CNS: the brain cortex, which undergoes dramatic morphogenetic changes, and the spinal cord that mainly grows by increase of volume. We will also cover the differences between cell states and cell fates, a current debate boosted with the latest large-scale molecular profiling studies. And finally, we will close discussing different hypotheses proposed to explain how progenitor cell diversity is generated and how they challenge prevailing concepts and raise new questions.

## Framing the Question: Progenitor Cells Versus Differentiated Neurons

The complex structure of our brain relies on the production and proper organization of diverse pools of neurons and glia from a relatively small number of neural progenitors during embryonic development. Despite the impressive progress in neurobiology over the last years, our understanding of how these multiple cell types are generated and maintained in highly organized spatial patterns, and how changes in this ground plan can result in pathologies, is still limited. We learnt that spatial patterning cues can produce different types of neural progenitors, and hence different types of neurons and glia along the anteroposterior (AP) or dorsoventral (DV) axes of the CNS (see section “The two paradigm models for tissue growth in the central nervous system: the spinal cord and the brain cortex”; Jessell, 2000). It is also known that neuronal production is asynchronous along the CNS, and work specially from Drosophila helped to unveil the molecular mechanisms by which individual progenitors sequentially generate the different cell types —a process called temporal cell specification (for review see Kohwi and Doe, 2013)—. Moreover, stem cells operate in a noisy and dynamic environment, as their gene expression levels fluctuate in response to intrinsic factors and/or environmental cues. Currently, big data approaches producing large amounts of measurements have the potential to help us to decipher how spatial and temporal cues are integrated to generate specific neuronal types and how aging progenitors change competence to produce different cell types over time.

The embryonic CNS is initially subdivided into regions along the body axes, where there is a progressive refinement of pattern. Each region has a distinct identity that underlies the generation of a specific set of cell types, each of which must be generated at the right time and place and in the correct proportions for normal development and proper function (Kiecker and Lumsden, 2005). The various neuronal populations found in the CNS arise from progenitor cells in specific locations of the embryonic neural tube. Moreover, cell diversity is generated at the same time that the brain undergoes a dramatic transformation from a simple tubular structure —the neural tube— to a highly convoluted structure —the brain, resulting in changes in the position of neuronal progenitors and their derivatives over time. In the developing CNS, the neural tube undergoes a segmentation process along the AP axis. This results in the formation of three embryonic brain vesicles —the forebrain, midbrain and hindbrain— and the elongated spinal cord (for review see Kiecker and Lumsden, 2005). At early stages of embryonic development, neuroepithelial cells (NEC) intensively proliferate by repeated symmetric cell divisions. NEC extend from the apical (ventricular) to the basal epithelial surfaces of the neural tube, and display interkinetic nuclear migration (IKNM) with the corresponding translocation of the nucleus according to the cell cycle phase –a beautiful orchestration between epithelial morphogenesis and cell proliferation. Although NEC are characterized by the expression of Sox2 and Nestin, and apical markers like Occludin and Zona Occludens 1 (ZO-1) (Götz and Huttner, 2005), there is no specific “molecular code” to define them. In spite of these common features, not all progenitors allocated in distinct CNS territories are equal. For instance, in the cortex, NEC gradually elongate and can become radial glial cells (RGC), with the cell bodies in the ventricular zone (VZ) and long radial fibers projecting to the basal surface. RGC undergo asymmetric cell divisions, giving rise to one RGC and either one immature neuron (IN) or an intermediate progenitor (IP). IP can further divide to give rise to neurons. Both NEC and RGC are considered as neural stem cells and are retained in the ventricular zone, close to the neural tube lumen (Alvarez-Buylla et al., 2001; Malatesta and Götz, 2013). They share the expression of several molecular markers and both cell types undergo IKNM (Than-Trong and Bally-Cuif, 2015). On the other hand, apical progenitors within the spinal cord retain NEC features and might proliferate symmetrically or asymmetrically, and this is ultimately governed by long-range morphogen gradients across the DV axis (Ulloa and Briscoe, 2007; Saade et al., 2013; Le Dréau et al., 2014). Thus, neural stem cells change their competency as development proceeds, and the generation of neuronal heterogeneity relies on the adscription of distinct progenitor/neurogenic competence (Beattie and Hippenmeyer, 2017). To acquire organs of a specific robust size and cell composition during development requires tight coordination between the maintenance of neural stem cells and the acquisition of neurogenic capacity. The rates of cell differentiation and proliferation must be tightly coordinated and balanced: early on, extensive cell proliferation dominates to allow the tissues to grow; later on, the growth rate ceases (or almost ceases, depending on the tissue) as more cells differentiate and exit the cell cycle (for review see Blanpain and Simons, 2013). Furthermore, and remarkably, stereotyped tissue growth must occur despite large variability in proliferation rates (He et al., 2012). If we consider tissues such as the CNS, in which differentiated neurons have no proliferation capacity, it implies that coordinating cell division modalities is crucial for regulating the growth of the tissue. Thus, symmetric self-renewing divisions for expanding the stem cell niche, asymmetric divisions for maintaining the progenitor pool —through this process stem cells are continually lost and replaced—, and finally either symmetric neurogenic divisions or direct cell differentiation need to be properly balanced to generate the right final number of differentiated neurons (for recent review see Zechner et al., 2020). This is accompanied with changes in the relative spatial distribution of both progenitors and differentiated neurons during morphogenesis. Clonal analyses, which describe the derivatives of a single cell, provide insight into the mode of tissue growth and its regionalization. They reveal the diversity of cell behaviors that underlies progression along a lineage tree, which has led to the elaboration of conceptual frameworks for cell lineage analysis (Buckingham and Meilhac, 2011). Thus, if we want to elucidate how the CNS is built up, we need to strengthen our knowledge about (i) the dynamics of the different cell populations (e.g., how progenitor cell populations spatiotemporally allocate, what the division rates are, and in what proportions), (ii) the transitions and switches between different division modes, and (iii) the sequential transition from the progenitor recruitment to the final functional neuronal populations.

During development, neural stem cells actively proliferate and give rise first to neurons, and then to glial cells. Neurogenesis is initiated by proneural genes, which encode basic helix-loop-helix (bHLH) transcription factors that form homodimers or heterodimers through the HLH domain and bind to DNA targets through the basic region (Bertrand et al., 2002). They trigger the specification of neuronal lineages and commit progenitors to neuronal differentiation by promoting cell cycle exit and activating a downstream cascade of differentiation genes (Castro et al., 2011; for review see Guillemot, 2007). The first step toward achieving the cell diversity observed in adults occurs with the organization of neuronal progenitor cells into distinct domains in response to morphogen signals. Such patterning signals drive the expression of specific sets of transcription factors and subdivide the developing nervous system into discrete progenitor domains (Ribes and Briscoe, 2009; Cohen et al., 2013) assigning spatial and molecular identity to them. The assigned identity depends on the location of the progenitors in the neural tube, and the interpretation of the two-dimensional grid, along the AP and DV axes. The transcription factors expressed in response to patterning signals will control the final neuronal fate. Once neuronal progenitors are committed, they undergo neuronal differentiation, migrating away from the ventricular zone, and giving rise to differentiated neurons. Thus, the spatiotemporal control of this process is fundamental for generating functional neuronal networks, and to ensure progenitor availability for later stages it is crucial to regulate their division mode, their quiescent state, and the timing at which distinct pools of progenitors engage in neurogenesis.

Addressing how spatiotemporally controlled cell proliferation, specification, and differentiation occur alongside morphogenesis in the CNS has been technically challenging to date; no *in vitro* system can recapitulate this *in vivo* process, which involves an extraordinary well-orchestrated migration of differentiated neurons from their birth site as well as complex tissue morphogenetic movements. Thus, reconstructing cell lineages has proved to be central to comprehend how the wide diversity of cell types is generated. Now, we have a wide palette of novel imaging and large-scale transcriptomic technologies to address this question. Next, we will briefly summarize them and discuss their advantages.

### From Cell Lineage to Cell Diversity: Genetically Encoded Lineage Tools

Intertwined with the concept of cell lineage is that of cell commitment. Cell lineage follows the normal fate of a cell and its daughters, leading to the formulation of genealogical trees of cells with increasingly restricted cell fate choices as development proceeds. For many years, comprehensive lineage reconstructions had been possible only in lower invertebrates, such as the nematode *Caenorhabditis elegans* (Sulston et al., 1983), or in basic chordates as *Ciona intestinalis* (Tassy et al., 2006). However, recent technological developments have proved to be valuable to address the lineages of organisms with non-stereotypic development. By reconstructing different cell lineages, we can now determine the functional cell transitions that distinct cell populations undergo, the impact of morphogenesis in the spatial distribution of progenitor cells, and the dynamics of the whole cell population. In other words, they provide the cellular data to complement the well-described GRNs involved in cell specification and differentiation. Multiple efforts have been deployed to developing tools for cell lineage analysis. These tools can be classified into: (i) cell birth-dating, aimed to identify when cells are born; (ii) cell fate mapping, to reveal the developmental potential of progenitor cells at later developmental stages; (iii) clonal analysis, to decipher the derivatives from a progenitor cell; and (iv) cell lineage tracing, to describe the mitotic connections between two or more genealogically related cells, allowing the assessment of cell lineages and cell behaviors in the whole organ context. They can be applied either to single cells in a mosaic manner or to an entire cell population (Garcia-Marques et al., 2021).

Imaging-based strategies provide an excellent spatial resolution, allowing to determine genetic clonal relationships based on the mitotic history, such as twin-spot Mosaic Analysis with a Repressible Cell Marker (twin-spot MARCM) in the nervous system of Drosophila (Yu et al., 2009), or Mosaic Analysis with Double Markers (MADM) in mice (Zong et al., 2005; Gao et al., 2014). The first enables the visualization of sister-paired clones from the same progenitor in two different colors, while the latest, permits to identify different recombination events by single or combined segregation of two fluorescent proteins (GFP and RFP) in daughter cells, therefore enabling the tracing of such derivatives in a total of three colors (green, red, and yellow). To improve their limited clonal resolution, multicolor labeling tools such as Confetti (Snippert et al., 2010), Brainbow (Livet et al., 2007; Weissman et al., 2011; Cai et al., 2013), StarTrack (García-Marqués and López-Mascaraque, 2013; Figueres-Oñate et al., 2016) or MAGIC (Loulier et al., 2014), rely on a stochastic and combinatorial expression of different fluorescent reporter genes induced by recombinases, which results in the generation of multiple color hues that label clonally related cells in the same color palette (for detailed reviews on cell lineage tools, both imaging and sequencing-based, see Espinosa-Medina et al., 2019; Figueres-Oñate et al., 2020; Garcia-Marques et al., 2021). Although the development of such multicolor strategies has been a major step forward in the cell lineage tracing field, they are not scalable, and in many cases they do not provide temporal resolution.

Noteworthy, the development of high-resolution 4D imaging paired with Light Sheet Fluorescence Microscopy (LSFM) using zebrafish transgenic embryos set up the path for understanding early embryonic development and assess cell lineages and behaviors at high spatiotemporal coverage and resolution (Keller et al., 2008; Olivier et al., 2010; Luengo-Oroz et al., 2011; Keller and Ahrens, 2015; Dyballa et al., 2017). This was accompanied with the development of cell-tracking tools, instrumental to reconstruct cell lineages and cell rearrangements upon time (Amat et al., 2014; Faure et al., 2016; Wolff et al., 2018; Wan et al., 2019). Although this approach provides valuable temporal information about cell lineages —and therefore cell hierarchies— in the context of the whole cell population, they are not scalable and need high computing power and specific *know-how* for the tracking analyses.

In addition to these imaging-based cell lineage tools, the development of CRISPR/Cas and the high-throughput and highspeed sequencing revolution have pushed forward the emergence of sequencing-based lineage strategies, which enable the establishment of cell connections upon unique genomic landmarks, also known as barcodes. Whether it is by Cas9/sgRNA induced genomic mutations (Cotterell et al., 2020; CARLIN, Bowling et al., 2020; ScarTrace, Alemany et al., 2018; LINNAEUS, Spanjaard et al., 2018) or by the insertion of exogenous arrays of DNA with multiple and inducible CRISPR/Cas target sites such as scGestalt (McKenna et al., 2016; Raj et al., 2018), barcoding tools label individual cells with a unique combination of scarred sequences. This cumulative stochastic barcode editing provides a unique DNA scar combinatory that will prevail in derivative cells, while adding up new generated ones. This enables the “tracing” of such derivatives after a transcriptomic analysis, using pseudo-time scales to generate cell trajectories and infer relationships between progenitor cells and their progeny. Similarly, other methods such as MEMOIR by engineered Mutagenesis with Optical *In situ* Readout, asses trajectories by combining barcoding elements and sequential rounds of multiplexed *in situ* hybridization (Frieda et al., 2017). Its improved version intMEMOIR goes further allowing the differentiation between cellular states due to the higher number of integrated barcode combinations as the result of the array’s inversion after genomic recombination (Chow et al., 2021). Although these strategies provide valuable single-cell transcriptional signature maps and atlases, the barcoding and omics combination still fails to represent cell behavior at the tissue level, neither provides cell division rates nor kinetics. Moreover, no functional relationships (circuits) are obtained.

To overcome such limitations, there is an urgent need for 4D tools that allow cell lineage relationships and temporality within the morphological context to be scalable. A few strategies have recently emerged that comply with the requirements of such need. One of these is CLADES (Cell Lineage Access Driven by an Edition Sequence), a genetic tool in Drosophila that enables cell lineage tracing coupled with birth-dating information. It is based on the sequential activation of a cascade of different reporters in progenitor cells by CRISPR/Cas9 induction, which are inherited by their differentiated progeny. This enables the cell lineage tracing without losing the temporal input, since early born and late-born cells will be labeled in different colors of the reporter cascade (Garcia-Marques et al., 2020). However, while clonal resolution is not an issue in Drosophila with a few and highly stereotypic lineages, cell lineages in vertebrates remain incompletely characterized due to the higher tissue complexity and the larger size of embryos. Therefore, there is still the demand to incorporate new strategies that couple temporal and clonal information preserving the anatomical context to fill the remaining gap between cell biology and genetic determinism. To strengthen the importance of such factors in a tissue context, in the next section we discuss how time and space shape differently two paradigmatic structures of the CNS, the brain cortex and the spinal cord.

## The Two Paradigm Models for Tissue Growth in the Central Nervous System: The Spinal Cord and the Brain Cortex

The CNS is comprised by morphologically different regions that become adult functional structures distinct in cell type composition and shape. Such structures are 4D developmental landscapes in which both the spatial coordinates and the temporal component are determining factors for the proper acquisition of cell types and numbers. Despite the impressive progress over the past decades, the comprehension of how billions of neurons come together to form the nervous system and enable function and behavior is still largely unknown. Two well-studied examples of intrinsically different structures of the CNS are the brain cortex and the spinal cord. While the first evolves from a relatively simple-layered neural tube to a complex structure with bulges and grooves, the oval embryonic spinal cord undergoes a volume scale-up with no dramatic change of form. Thus, the requirements for coordinating cell specification and morphogenesis are expected to differ. In this section, we contrast the biology of both paradigms and discuss several studies that demonstrated the role of cell position and time on cell specification, cell fate, and tissue growth.

### Position (and Time) Determines Neural Identity and Growth in the Spinal Cord

The characterization of the adult spinal cord according to morphology, molecular markers, neuronal connectivity and axonal projections revealed a modular organization with stereotypical position of specific neurons (Sagner and Briscoe, 2019). During spinal cord formation, long-range morphogen signals emanating from the roof and floor plates pattern the tissue along the DV axis by regulating cell fate through transcription factor expression. This transcription factor code defines 11 molecularly distinct neural progenitor domains –six dorsal and five ventral–, each of which gives rise to one or more different neuronal subtypes (Alaynick et al., 2011; Lu et al., 2015). As development proceeds, progenitors in each domain specify in a spatiotemporally ordered manner and either amplify, or give rise to the corresponding type of post-mitotic neurons (Jessell, 2000; Dessaud et al., 2008; Le Dréau and Martí, 2013). Thus, the stereotypical position of cells –and the spatial regulation of gene expression– is crucial in the formation of neuronal circuits. However, several large-scale molecular profiling studies provided catalogs of gene expression, revealing a higher complexity of cell types (Delile et al., 2019; Rayon and Briscoe, 2021). For instance, single-cell RNA-sequencing experiments in mice embryonic and adult spinal cord suggest the existence of at least several dozen of molecularly different neuronal subtypes (Delile et al., 2019). This reveals the sequential upregulation or induction of sets of transcriptions factors that underpin the identity of the derivative arising neurons, generating a temporal stratification of neuronal subtypes from each domain. Thus, complementary to the positioning, time also plays a role in neuronal identity acquisition and in the generation of neuronal diversity (Sagner and Briscoe, 2019).

In the spinal cord, distance from the DV poles seems to dictate progenitor competence since neural progenitors acquire distinct identities in response to opposing morphogen gradients (Jessell, 2000; Ribes and Briscoe, 2009; Le Dréau and Martí, 2013). These morphogens, Sonic hedgehog (Shh) ventrally and Bone Morphogenetic Proteins (BMP) and Wnts dorsally, induce the expression of several homeodomain and bHLH transcription factors in discrete domains along the DV axis (Kutejova et al., 2016). Since their combinatorial expression confers neuronal identity to progenitors, they are exquisitely regulated. They cross-regulate forming a well-defined GRN accountable for the response to morphogen gradients by modular enhancers (Peterson et al., 2012; Oosterveen et al., 2013). During neurogenesis, the spinal cord continues to grow along its DV axis and expand the mantle zone with the differentiated neurons, raising the questions of how discrete progenitor domains and specific gene expression territories remain stable and scalable upon being challenged by cell proliferation and how DV patterning and neurogenesis are intertwined. Lately, a two-phase model has been proposed for explaining the growth and patterning of the spinal cord (Kicheva and Briscoe, 2015). The pattern of neuronal progenitor domains would be established at early developmental stages, when the position of a cell within the morphogen gradients grid can be precisely decoded (Sagner and Briscoe, 2019). These progenitors would maintain certain cell plasticity such as they could switch identities (Dessaud et al., 2007, 2010), facilitating the transition along different progenitor states. Upon tissue growth, the pattern of progenitor domains would be maintained by GRN cross-repressive interactions and unequal neuronal differentiation rates would determine domain sizes (Kicheva et al., 2014). However, how the neuronal differentiation dynamics of different progenitor populations is regulated has not been revealed. Interestingly, the regulatory programs and neuronal cell types are highly similar in different vertebrates, despite the distinct developmental time scales across species. Differences in protein turnover play a role in interspecies differences in the tempo of motoneuron differentiation (Rayon et al., 2020); however, whether similar mechanisms may operate to specifically regulate the differentiation rate of the distinct progenitor domains is not known yet.

Dynamics of morphogen signaling and cell division mode have been linked in the spinal cord, since the onset of neurogenesis in dorsal interneurons and ventral motoneurons is controlled by BMP/SMAD- and Shh-signaling, respectively (Saade et al., 2013; Le Dréau et al., 2014). As example, in motoneuron progenitors (MNp) Shh maintains self-expanding symmetric proliferative divisions, while preventing progenitors from switching to neurogenic divisions. A reduction in Shh activity results in reduction of symmetric proliferative cell divisions, coinciding with the developmental time of motoneuron generation (Saade et al., 2013, 2017). While clones of the MNp domain grow equally in both axes, the rest of the domains show more elongated cell clones in DV, resulting in an inferior net growth rate DV/AP (Kicheva et al., 2014). A 3D computational simulation of the spinal cord DV growth shows that the differences in the spread and shape of MNp clones, and the isotropic growth, can be explained by the higher differentiation rate of these progenitors (Kicheva et al., 2014; Guerrero et al., 2019). Overall, these studies demonstrate that DV progenitor position influences proliferative capacity, cell fate and growth in the spinal cord. However, they do not explain why MNp differentiate at a higher rate than progenitors in adjacent domains. As neurons differentiate, they delaminate toward the mantle zone. This active displacement of neurons shapes the spinal cord in such a manner that progenitor cells are kept in the ventricular zone and differentiated neurons allocate in the adjacent medial domain, and the tissue grows without dramatic morphogenetic changes.

In several systems temporal cues regulate neuroblast competence –and therefore expansion of neural diversity– by specifying distinct neuronal fates using combinatorial temporal patterning (Bayraktar and Doe, 2013). In the spinal cord, the birth order of neurons also underlies specificity in neuronal connectivity and circuit formation (McArthur and Fetcho, 2017; Pujala and Koyama, 2019; Wan et al., 2019). As previously mentioned, several works have stressed the importance of the temporal transcription factor code for subdividing neurons through the DV axis (Delile et al., 2019). Although these results suggest that the temporal transcriptional factor code is functionally important, it seems that temporal cues would work within a given neuronal population to help to expand its diversity. Thus in the spinal cord, the precise position of neural progenitors serves as a functional ground for neuronal subtype determination.

Similar to other regions of the CNS, neural progenitors in the spinal cord give rise first to neurons and later to glial cells. This temporal switch relies on the sequential induction of SoxE and NFI factors and is regulated by several signaling pathways (Deneen et al., 2006; Kang et al., 2012). As an example, studies in zebrafish embryos demonstrate that motoneurons and oligodendrocytes emerge from the same ventral progenitor domain, the MNp (Zannino and Appel, 2009; Esain et al., 2010). In mice, most of the oligodendrocytes are generated after motoneurons in a Shh-dependent manner (Soula et al., 2001; Fogarty et al., 2005). However, 5% of the total population arises in a Shh-independent manner from a dorsal Dbx1-expressing region at early postnatal stages and distribute to the lateral white matter, radially opposite to their site of origin. In contrast, pMN-derived oligodendrocyte cells usually distribute in the gray matter (Fogarty et al., 2005). Similarly, DV position also determines the astrocytic subtype since the expression of *Pax6* and *Nkx6.1* confers positional identity defining three distinct astrocyte subpopulations. Each of these progenitor domains displays a specific code for Reelin and Slit guidance molecules, resulting in a correlation between the origin of astrocyte subtype and their final position within the neural tube (Hochstim et al., 2008). Thus, in oligodendrocytes and astrocytes, both birth-dating and DV position within the spinal cord influence their final location within the adult structure.

### Time Determines Neural Identity and Growth in the Brain Cortex

Since Cajal’s descriptions of the brain cortex cytoarchitecture and laminar distribution, the development of clonal analysis and cell lineage tools has fastened and accurately unveiled its organization and composition. The cerebral cortex evolves from a dense and packed single cell sheet composed solely by progenitors –the embryonic forebrain– to a stratified tissue remarkably conserved across most mammals. The neocortex is organized into six distinct layers, each of them with neuronal heterogeneity that emerges from sequentially born progenitors. The ventricular zone (VZ) harbors the soma of progenitor cells, followed by the subventricular zone (SVZ) as the main area of cell amplification. The cortical plate consists of several cell layers that sequentially accumulate on top (LI-LVI, being LI the uppermost and LVI the deepest layer), in an ‘inside-out’ manner, where early born neurons locate in deep layers, whereas newly born neurons migrate and position in upper layers (Angevine and Sidman, 1961; Rakic, 1974; Takahashi et al., 1999). In contrast, their glial counterparts organize in a stochastic manner along the apicobasal extent of the cortical plate (Zhang X. et al., 2020).

Neuroepithelial cells are the early progenitors populating the VZ, which divide in a symmetric proliferative manner prior to neurogenesis (Subramanian et al., 2017). As neurogenesis starts, NEC transition to RGC that are classically defined by the combination of several features: (i) an elongated morphology with contacts in the apical and basal surfaces of the neuroepithelium; (ii) the maintenance of the apicobasal polarity, (iii) the expression of astroglial markers such as glutamate transporter (GLAST) (Hartfuss et al., 2001), glial fibrillary acidic protein (GFAP), glutamine synthase (GS), and brain-lipid-binding protein (BLBP) (Feng et al., 1994; Arellano et al., 2021). Nascent RGC may undergo symmetric proliferative cell divisions to expand the progenitor pool, and later they transition into the neurogenic state and asymmetrically divide, thereby producing a self-renewed RGC and a differentiated cortical neuron (Noctor et al., 2004). Ventricular RGC might as well give rise to one RGC and either an IP or to another progenitor type, the basal radial glial cell (bRGC) (Miyata et al., 2001; Haubensak et al., 2004; Noctor et al., 2004) that migrates to the SVZ becoming the major contributor to neuronal diversification (for reviews see Huttner and Kosodo, 2005; Penisson et al., 2019). These bRGC differ from apical RGC in their retraction of the ventricular processes before their division (Miyata et al., 2001), and in their division mode, since they usually generate either two bRGC by symmetric proliferative division or two daughter neurons by symmetric differentiative division (Haubensak et al., 2004; Noctor et al., 2004).

By consecutive waves of neurogenesis, distinct cortical layers are formed in the ‘inside-out’ fashion (for reviews see Rakic, 2009; Taverna et al., 2014; Hansen et al., 2017). Despite its pluripotency, cortical RGC undergo a progressive fate restriction over time (Desai and Mcconnell, 2000), since they lose the capacity to generate deep cortical layer neurons, limiting their derivatives to upper layers (McConnell and Kaznowski, 1991; Frantz and McConnell, 1996). Most clonal analysis studies suggest that the RGC behavior can be predictable across all developmental stages. RGC in the neurogenic phase do not undergo terminal differentiation in a stochastic manner but rather follow a defined non-random program of cell cycle exit resulting in eight to nine neurons produced by one RGC (Gao et al., 2014). In the same line, MADM clones induced in RGC at later developmental stages, right before the onset of gliogenesis, show more neurons in the upper layers (Zhang X. et al., 2020). Similarly, other studies demonstrate that as more fate restricted the cortex progenitor cells are, less neuronal cell types they are able to generate. For instance, when IP are early targeted, the derived neurons locate mainly in deeper layers instead of covering the entire translaminar area (Mihalas and Hevner, 2018); when they are targeted even later in development, they mainly produce neurons that locate in upper layers instead (Tarabykin et al., 2001). These results indicate that RGC constitute a pretty homogeneous cell population. However, a recent report shows that a limited number of progenitors display a stochastic neuronal output to account for the diverse clone types (Llorca et al., 2019). When they map the lineage of genetically labeled progenitor cells focusing on progenitors that start generating neurons early during development, they observe that early born neurons locate in deep layers as expected, and that a substantial group of neurons are confined either to the deep or superficial layers. They propose that heterogeneous lineage configurations can arise directly from neurogenesis and contribute to diverse neuronal types (Llorca et al., 2019). Overall, these observations suggest that the laminar position allows a crude classification of projection neurons and dictates their connectivity, although the progenitor population might not be so homogeneous.

Radial glial cells can also produce glial cells, both astrocytes and oligodendrocytes, which organize dispersedly along the apicobasal extent of the cortical plate. Once the neurogenic capacity of the remaining progenitors decreases, intrinsic and extrinsic signals set the start of gliogenesis (for review see Kessaris et al., 2008). However, the mechanisms of lineage progression from neurogenesis to gliogenesis remain largely unexplored. Astrocytes arise few days later than neurons as the result of remaining RGC detaching from apicobasal poles and retracting their projections (Noctor et al., 2004). Although astrocytes show some layer-specific features (Lanjakornsiripan et al., 2018; Batiuk et al., 2020), a clonal analysis study using MAGIC Markers combinatorial labeling, demonstrates that astrocytes do not follow an ‘inside-out’ pattern; instead, they distribute along the cortical area and acquire their fate in a stochastic manner (Clavreul et al., 2019; Zhang X. et al., 2020). It is well known that oligodendrocytes arise from both NG2-positive (Mo and Zecevic, 2009) and NG2-negative (Gensert and Goldman, 2001) oligodendrocyte progenitor cells; however their birth-date is still unclear. There are several reasons for this such as (i) oligodendrocytes found in the cortex are a result of competing waves emanating both locally and from other brain areas (Spassky et al., 1998; He et al., 2001; Tekki-Kessaris et al., 2001; Gorski et al., 2002; Kessaris et al., 2006), and (ii) fully differentiated and functional myelinating oligodendrocytes maturate during postnatal stages (for reviews on intrinsic and extrinsic factors driving oligodendrocyte development and maturation see Meijer et al., 2012; Baydyuk et al., 2020). Neuronal layer inversion studies suggest oligodendrocytes indeed need the correct sequential positioning of neurons to acquire their characteristic asymmetric distribution along the cortical area (Tan et al., 2009). Thus, temporal cues regulate the successive generation of layered postmitotic neurons and glial cells. Although we have a framework for RGC lineage progression, there are still open questions such as (i) how heterogenous the pool of RGC is, (ii) whether deterministic and stochastic modes of neuronal production coexist, and (iii) how cortex morphogenesis and cell fate acquisition are coordinated.

**FIGURE 1:**
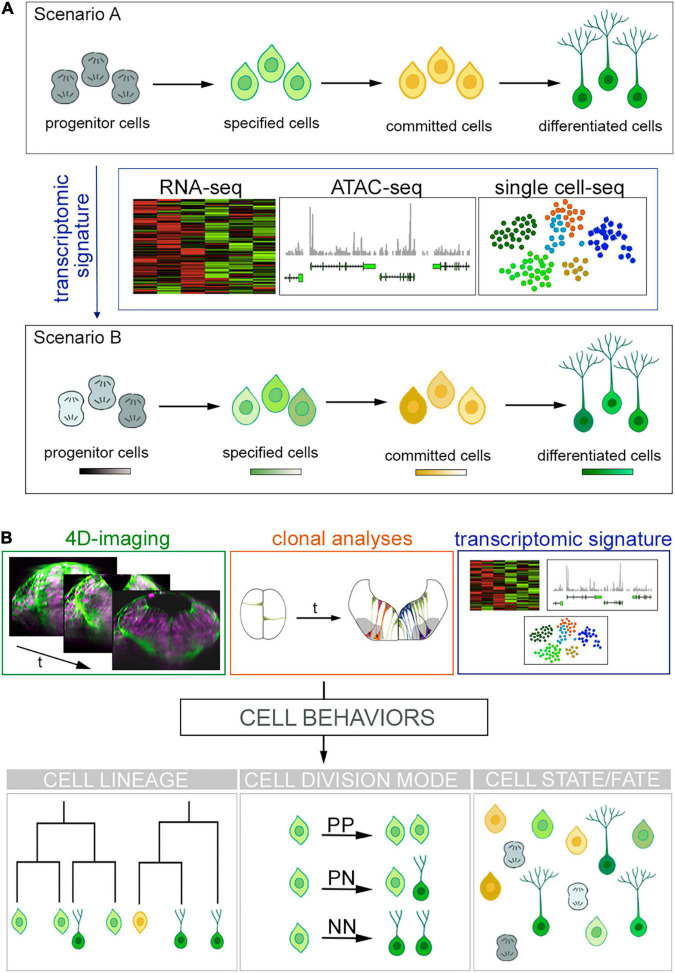
Cell fates vs. cell states and new approaches for cell lineage reconstruction. **(A)** Overview of the current scenarios for cells progressing toward differentiation. Scenario A shows progenitor cells transitioning toward specification and commitment to finally differentiate. New high-throughput sequencing technologies (RNA-seq, ATAC-seq, and single cell sequencing) produce large volumes of data providing transcriptomic signatures. This unveils the emergence of different cell states within a cell fate depicted as color hues in Scenario B. **(B)** Future challenges to comprehend how cells acquire their fate. Next generation tools might blend big data provided by (i) 4D imaging that informs us about cell hierarchies and behaviors (see transverse views of zebrafish hindbrain over time; cell nuclei are in magenta and plasma membranes in green), (ii) clonal analyses, which informs about tissue growth; and (iii) transcriptomic signatures telling us about GRN in order to fill the gap between genetic determinism, cell behavior and cell fate while keeping the morphological context. The best scenario would be to blend such amount information in order to understand cell behaviors and to generate full cell lineages.

Recent advances have challenged that time and spatial location are the main determining factors for cell specification and cell fate acquisition for the generation of cell diversity in the CNS. High-throughput transcriptional profiling studies have allowed the envisioning of new horizons for cell characterization based on their individual RNA profile and challenged the “cell fate” concept proposing cellular state as the accurate terminology (Figure 1A). This brings on the table an open debate that goes beyond nomenclature: cell fate or cell state?

## Cell Fate Versus Cell State: The Never-Ending Debate

In recent years, new powerful and high-resolution methods such as single-cell transcriptomics and single-cell barcoding lineage tracing have challenged the classical lineage tree view, where stem cells have unlimited potential and each of the multiple progenitor populations have a predetermined fate. It is becoming increasingly apparent that cells have the bias toward a certain fate, while progenitor populations display certain plasticity. Therefore, the idea of a differentiation tree in which the stem and progenitor populations are separated and differentiation occurs as discrete steps along the tree is changing to a model where differentiation is a continuous process, with stem and progenitor cells being biased toward a certain fate. Currently, this is extensively debated in the hematopoietic system, which has long served as a model for stem-cell research (Laurenti and Göttgens, 2018), and it is suggested that this scenario could be shared in the CNS. Indeed, broad sampling of different CNS territories using single-cell transcriptional profiling allows us to monitor global gene expression in thousands of individual cells. This enables the identification of wider progenitor cell types than previously recognized, and provides an extraordinary molecular characterization. The use of these big data approaches and the ability of integrating them with the cellular and genomic data, are the challenges to overcome in order to transform the biological knowledge into useful insights for treating the neurological disorders (Briscoe and Marín, 2020).

### Cell Fate Versus Cell State of Neural Cells

As previously stated, new technological approaches mainly based in single-cell molecular profiling elicit new arguments about what a cell type is. Cell types are the basic building blocks of multicellular organisms, determined and maintained by gene regulatory programs; however, cell type classification schemes remain ambiguous (Arendt et al., 2016). A classic discrete cell type categorization from progenitor cells to their differentiated derivates has been challenged lately as such description considers that cells follow discrete steps in a linear path to acquire their fate. Instead, it has been suggested cells navigate through different states toward differentiation (Figure 1A). Conceptually, cell fate comprises the future identity of a cell and it is determined by multiple factors such as gene expression, cell–cell interactions and external cues –both mechanical and biochemical. Therefore, cell fate would be the discrete and final step that defines the type of a cell. In contrast, the state of a cell implies a temporary feature, as it undergoes transitions over time from a starting point in space to the next one in a continuum of a dynamic system. Such scenario is usually envisioned as a space of states (Trapnell, 2015) and has recently become one of the most fervent debates in biology (see Clevers et al., 2017 for disparate views on cell identity, cell fate and cell state concepts).

Although a cell type is characterized by morphology, function, position and gene expression, even homogeneous cell type populations display high heterogeneity in their transcriptional profile. The current challenges biology faces are the untangling and the interpretation of this heterogeneity at the individual cell level, and for this, many genetic and transcriptomic profiles are carried out to characterize individual cell signatures. Posterior cell clustering and mapping of such libraries identify intermediate cell states, as single cells with a similar transcriptomic profile are likely to be closely related. However, the question poses: can we predict from the transcriptome of a progenitor cell, the identity, connectivity and function of their derivatives?

Several studies have reported the existence of transitory cell states within the CNS using these approaches. For instance, single-cell RNA-sequencing analysis of FACS sorted cortical apical progenitors identifies different transcriptional states according to the pseudo-developmental stage (Telley et al., 2019). While at early mice embryonic stages apical progenitors are characterized by the expression of genes related with cell intrinsic transcriptional programs, at later stages they progress to a more environmental-sensing transcriptomic signature, which already suggests that environmental cues may play a key role in refining the neuronal heterogeneity arising from cortical apical progenitors (Telley et al., 2019). In the zebrafish hindbrain, a single-cell RNA-sequencing study at different embryonic patterning stages identified discrete cellular states that differ on their transcription factor expression, and recapitulate the transition of progenitors to neuronal differentiation (Tambalo et al., 2020). This can suggest that cells display different competence states, and/or the existence of a heterogeneous progenitor pool. Barcoding systems have contributed exponentially to the untangling of new cellular states in the CNS by generating cell trajectories (Raj et al., 2018), although in none of the cases cell hierarchies could be established. As we discussed in Section “Framing the question: progenitor cells vs. differentiated neurons” of this review, the impact of single-cell transcriptomics on the characterization of gene programs for neuronal diversification is undeniable; however, these tools are based solely on transcriptomic signatures with neither spatial organization nor cell–cell contact inputs since they require tissue dissociation. Moreover, since they rely on pseudo-time parameters they lack the developmental history of cells, raising the question of whether cell identity can be defined by a single signature pattern of gene expression. Although the integration of imaging approaches that maintain the 3D tissue conformation might solve the first issue, such as MERFISH (Chen et al., 2015; Zhang M. et al., 2020) or STARmap (Wang et al., 2018), these trajectory-based assays alone are not sufficient to capture the intricacy of such dynamic systems.

These high-resolution data approaches provide an unprecedented level of detail and are indeed revolutionizing the study of CNS development. They demonstrate (again) that complexity is build up during embryonic development, and suggest that once “crude cell fates” are established, the final cell identities are refined upon time with cells transitioning through different cell states. In other epithelial systems, stem cells constitute a heterogeneous compartment in which cells transit reversibly between different states of competence (Blanpain and Simons, 2013). The big leap forward would be to combine cell lineage, developmental cell trajectories, and molecular mechanisms, to comprehend how neuronal diversity arises. Most importantly perhaps, if dynamic cellular features are predictable at a population rather than single-cell level, understanding the emergent properties of cell populations instead of by the detailed account of their individual components should be considered to address the emergence of functional circuits during embryogenesis.

#### Pools of Progenitors and Quiescent Stem Cells

If this scenario was not complex enough, the CNS harbors groups of cells that display a different progenitor behavior, such as (i) progenitor pools that engage into neurogenesis at different times, and (ii) quiescent progenitor cells, which are out of the cell cycle progression and more commonly found in adult stages. Their presence and long-term maintenance are crucial for the acquisition of tissue cell diversity, survival and regeneration after injury (for reviews on quiescence see Cheung and Rando, 2013; Cho et al., 2019; van Velthoven and Rando, 2019). Interestingly, such differences between progenitors are not always evident at a molecular level, thus, the transcriptional signature becomes a powerful distinction tool to assess state differences. Although usually considered dormant, quiescent cells require an active and complex regulation, thus, considering quiescent or active neural stem cells as binary fates or binary cell states is incorrect. In fact, there is a gray scale of states in which the so called primed neural stem cells –in a less deep quiescent state– are able to revert their “dormancy” to activate and contribute to adult neurogenesis, being able to return to quiescence after their contribution (Sueda et al., 2019; Urbán and Cheung, 2021).

In the mouse forebrain, for instance, barcoding studies revealed that progenitors in a dormant state upregulate genes related with their ability to keep in their adult quiescent niche, which differentiate them from their active counterparts. Also, although quiescent progenitors from different forebrain regions maintain the transcriptional hallmarks of their specific embryonic ancestor cells, once they reacquire an active state they cluster together showing a similar transcriptomic signature and favoring the same neuronal type production (Borrett et al., 2020). This illustrates that different cellular transitory states might result in the same final fate. Such transcriptional hallmarks acquired during embryogenesis and shared at later developmental stages, have also been reported in the zebrafish brain (Raj et al., 2018). On a similar note, a clonal analysis approach demonstrates that adult neural stem cells of the mouse cortex arise from progenitors already specified at early embryonic stages (Fuentealba et al., 2015; Furutachi et al., 2015). These results raise some inevitable questions: is there such thing as a progenitor fate? If we understand fate as the final state of a cell, and considering quiescent cells might eventually reactivate upon environmental requirements, one could argue that in this case there is no such thing as a progenitor fate and the terminology “steady-state” might be more accurate. On top of that, the previously mentioned evidences on cell trajectories and cell lineage tracing, linking progenitor cells with adult quiescent neural stem cells at a transcriptomic and molecular levels, denote a putative cell fate or “steady-state” determination from birth. What brings another question for open discussion: is cell fate determined, or is this a stochastic process?

### Cell Fate Decisions: Stochasticity Versus Determinism

A long-standing question in developmental neurobiology is to determine how cell diversity arises from groups of “equivalent” progenitor cells. Understanding how cell fate choices are made is crucial to comprehend the spatiotemporal dynamics of tissue and organ formation and to predict cell behaviors. Waddington’s analogy of cells represented as bowls rolling down valleys in a downhill landscape is one of the most well-known examples of cell fate decision-making in a dynamic system (Waddington, 1957). The path to two different states or fates is illustrated as branching valleys and it represents fate choices in a cell’s endeavor, not mitotic events. This model, referred as well as *the epigenetic landscape*, proposes that a cell’s potential for development, meaning the down-path it takes, is marked by groups of genes or biochemical interactions, already speculating about the GRN control of cell fate decision (for reviews see Enver et al., 2009; Fagan, 2012). Waddington’s landscape model is general, qualitative, and although portraits the concept of cell competence, it does not provide the 3D-positional input within the tissue, or it considers intermediate cell states between one decision and the next. It implies bowls (cells) that go down chosen valleys (fate decision) cannot go back to the previous position once they are committed. It does not resolve if the decision of choosing a developmental path (to go through the different valleys) is stochastic or already determined. Since Waddington’s theory, several genetic fate mapping studies in combination with mathematical models have tried to tackle this issue in specific structures (for review see Zechner et al., 2020). While there is no debate around the vast heterogeneity harbored in the CNS, there is not a single view in the generation and acquisition of such cell type variety. While some lines of research suggest it must correlate with progenitor subtype diversity –implicating fate is determined before differentiation–, other studies suggest that the brain may harbor multipotent progenitors whose decision of generating different fates is merely stochastic.

One of the best studied cases is the brain cortex (see section “The two paradigm models for tissue growth in the central nervous system: the spinal cord and the brain cortex”). In the recent years the technological advances in single-cell transcriptomes led to revisit the knowledge on cortical projection neuron heterogeneity and its ontogeny (Briscoe and Marín, 2020). A genetic fate mapping study on a subset of RGC demonstrates that the murine cerebral cortex contains RGC sub-lineages with distinct fate potentials. Using *in vivo* genetic fate mapping and *in vitro* clonal analysis, they identify a Cux2-positive RGC lineage intrinsically specified to generate only upper-layer neurons, independently of niche and birthdate. Interestingly, when forced to exit earlier the cell cycle, the outcome is also specific for upper layer neurons, indicating these RGC progenitors already specified to generate upper layer neurons regardless of birthdate were intrinsically programmed to generate neurons predominantly later than their lower layer counterparts (Franco et al., 2012). Thus, this study indicates that molecular fate specification ensures proper birth order, rather than vice versa. In this same line, a MADM clonal analysis on RGC progenitors revealed that all progenitors give rise to eight to nine neurons in a reproducible way, and that after this stereotyped neuronal generation, glial cells are produced. Such behavior was interpreted as a deterministic neural fate acquisition pattern (Gao et al., 2014). However, Guo et al. (2013) demonstrated the existence of multipotent neocortical progenitors. Genetic fate mapping of Cux2-positive RGC shows that they sequentially generate both deep- and upper-layer projection neuron subtypes and glia. More recently, clonal analysis studies combined with mathematical models favored the existence of a stochastic acquisition of neuronal fates, challenging again the deterministic view. They developed mathematical models that could emulate their biological observations in mice cortex using MADM-induced clonal analysis, and the best fitting models suggested that indeed neurogenic fate decisions could be stochastic (Llorca et al., 2019). They proposed that the heterogeneity of neuronal fates in the cortex might be explained by the existence of two distinct progenitor cell populations, which would randomly generate the translaminar cell diversity across the cortical plate. These observations raise further questions such as (i) can the two progenitor subtypes be molecularly identified, (ii) do the stochastic events occur within progenitors or in the progeny, and (iii) what is the relative final contribution of stochastic and deterministic processes (Klingler and Jabaudon, 2020).

The stochasticity in neurogenic fate decisions has also been shown in other CNS structures such as the adult zebrafish telencephalon (Than-Trong et al., 2020) and both zebrafish and *Drosophila*’s retina (Bell et al., 2007; He et al., 2012). Intriguingly, both cell fate decision mechanisms operate in the retina. Retinal progenitor cells have the same competence; however, extrinsic and intrinsic cues induce cell fate in a reproducible deterministic manner during embryonic development (for review see Cepko, 2014). Stochastic cell fate decisions are most abundant at early stages of retinal neurogenesis (He et al., 2012). By contrast, more deterministic division patterns become prominent late in embryonic development, probably because they often arise from a committed precursor, which gives rise to later born neurons. For instance, in zebrafish the assignment of Müller glial fate, a retina-specific RGC, has been shown to follow a deterministic pattern instead (Rulands et al., 2018). This is a clear illustration of how two distinct neural fates can be acquired following a different pattern within the same structure. Overall, these data suggest that generation of cell diversity cannot be explained by one model solely but instead might be better represented by a synergistic cooperation of stochastic and deterministic features.

## Future Challenges

New technologies and large datasets are providing new perspectives on long-standing questions about the ontogeny, the composition, and the function of the cellular components of the CNS. However, the progress will depend not only on the improvement of acquisition and analytical capacity to process big amounts of data, but on their successful application to build up intellectual frameworks (Figure 1B). For the question of cell types/cell identities and cell fates/cell states, the prevailing view is that each type of neurons uses a specific set of features such as gene expression, morphology, position, neuronal activity, … to define cell identity, which are regulated by specific transcriptional signatures. This would be consistent with the idea that cell identity is defined by the specific gene expression programs executed by GRN (Davidson and Erwin, 2006). But is the knowledge of a neuron transcriptome sufficient to define its identity and predict the functional features? Probably not if we consider the role of cell hierarchies, and that morphologically different neurons located in either distinct or similar regions of the CNS can be transcriptomically similar. For instance, motoneurons from the hindbrain and the spinal cord are quite similar in terms of gene expression, but their ontology is different. Thus, cell lineage –and therefore the temporal component– is also likely to be an important feature to comprehend what cell type identity means. Can we reconstruct neurogenesis from birth to entire circuit at cell type and functional levels? Can we monitor the emergence of coordinated neuronal activity at single-cell level and see how circuits are build up upon development? Can we apply this knowledge to brain organoids derived from human iPS to mimic the spatial and temporal developmental landscapes for easier manipulation? And finally, can we create organs upon demand to substitute parts of the old ones?

## Author Contributions

CB-M and CP contributed to the concept, design, and writing of the review. Both authors contributed to the article and approved the submitted version.

## Conflict of Interest

The authors declare that the research was conducted in the absence of any commercial or financial relationships that could be construed as a potential conflict of interest.

## Publisher’s Note

All claims expressed in this article are solely those of the authors and do not necessarily represent those of their affiliated organizations, or those of the publisher, the editors and the reviewers. Any product that may be evaluated in this article, or claim that may be made by its manufacturer, is not guaranteed or endorsed by the publisher.

## References

[B1] Alaynick W. A. Jessell T. M. Pfaff S. L. (2011). SnapShot: spinal cord development. *Cell* 146 1–7. 10.1016/j.cell.2011.06.038 21729788 PMC3158655

[B2] Alemany A. Florescu M. Baron C. S. Peterson-Maduro J. Van Oudenaarden A. (2018). Whole-organism clone tracing using single-cell sequencing. *Nature* 556 108–112. 10.1038/nature25969 29590089

[B3] Alvarez-Buylla A. García-Verdugo J. M. Tramontin A. D. (2001). A unified hypothesis on the lineage of neural stem cells. *Nat. Rev. Neurosci.* 2 287–293. 10.1038/35067582 11283751

[B4] Amat F. Lemon W. Mossing D. P. McDole K. Wan Y. Branson K. (2014). Fast, accurate reconstruction of cell lineages from large-scale fluorescence microscopy data. *Nat. Methods* 11 951–958. 10.1038/nmeth.3036 25042785

[B5] Angevine J. B. Sidman R. L. (1961). Autoradiographic study of cell migration during histogenesis of cerebral cortex in the mouse. *Nature* 192 766–768. 10.1038/192766b0 17533671

[B6] Arellano J. I. Morozov Y. M. Micali N. Rakic P. (2021). Radial glial cells: new views on old questions. *Neurochem. Res.* 46 2512–2524. 10.1007/s11064-021-03296-z 33725233 PMC8855517

[B7] Arendt D. Musser J. M. Baker C. V. H. Bergman A. Cepko C. Erwin D. H. (2016). The origin and evolution of cell types. *Nat. Rev. Genet.* 17 744–757. 10.1038/nrg.2016.127 27818507

[B8] Batiuk M. Y. Martirosyan A. Wahis J. de Vin F. Marneffe C. Kusserow C. (2020). Identification of region-specific astrocyte subtypes at single cell resolution. *Nat. Commun.* 11 1–15. 10.1038/s41467-019-14198-8 32139688 PMC7058027

[B9] Baydyuk M. Morrison V. E. Gross P. S. Huang J. K. (2020). Extrinsic factors driving oligodendrocyte lineage cell progression in CNS development and injury. *Neurochem. Res.* 45 630–642. 10.1007/s11064-020-02967-7 31997102 PMC7058689

[B10] Bayraktar O. A. Doe C. Q. (2013). Combinatorial temporal patterning in progenitors expands neural diversity. *Nature* 498 449–455. 10.1038/nature12266 23783519 PMC3941985

[B11] Beattie R. Hippenmeyer S. (2017). Mechanisms of radial glia progenitor cell lineage progression. *FEBS Lett.* 591 3993–4008. 10.1002/1873-3468.12906 29121403 PMC5765500

[B12] Bell M. L. Earl J. B. Britt S. G. (2007). Two types of *Drosophila* R7 photoreceptor cells are arranged randomly: a model for stochastic cell-fate determination. *J. Comp. Neurol.* 502 75–85. 10.1002/cne.21298 17335038

[B13] Bertrand N. Castro D. S. Guillemot F. (2002). Proneural genes and the specification of neural cell types. *Nat. Rev. Neurosci.* 3 517–530. 10.1038/nrn874 12094208

[B14] Blanpain C. Simons B. D. (2013). Unravelling stem cell dynamics by lineage tracing. *Nat. Rev. Mol. Cell Biol.* 14 489–502. 10.1038/nrm3625 23860235

[B15] Borrett M. J. Innes B. T. Jeong D. Tahmasian N. Storer M. A. Bader G. D. (2020). Single-cell profiling shows murine forebrain neural stem cells reacquire a developmental state when activated for adult neurogenesis. *Cell Rep.* 32:108022. 10.1016/j.celrep.2020.108022 32783944

[B16] Bowling S. Sritharan D. Osorio F. G. Nguyen M. Cheung P. Rodriguez-Fraticelli A. (2020). An engineered CRISPR-Cas9 mouse line for simultaneous readout of lineage histories and gene expression profiles in single cells. *Cell* 181 1693–1694. 10.1016/j.cell.2020.06.018 32589959 PMC7530571

[B17] Briscoe J. Marín O. (2020). Looking at neurodevelopment through a big data lens. *Science* 369:eaaz8627. 10.1126/SCIENCE.AAZ8627 32943499

[B18] Buckingham M. E. Meilhac S. M. (2011). Tracing cells for tracking cell lineage and clonal behavior. *Dev. Cell* 21 394–409. 10.1016/j.devcel.2011.07.019 21920310

[B19] Cai D. Cohen K. B. Luo T. Lichtman J. W. Sanes J. R. (2013). Improved tools for the brainbow toolbox. *Nat. Methods* 10 540–547. 10.1038/nmeth.2450 23817127 PMC3713494

[B20] Castro D. S. Martynoga B. Parras C. Ramesh V. Pacary E. Johnston C. (2011). A novel function of the proneural factor Ascl1 in progenitor proliferation identified by genome-wide characterization of its targets. *Genes Dev.* 25 930–945. 10.1101/gad.627811 21536733 PMC3084027

[B21] Cepko C. (2014). Intrinsically different retinal progenitor cells produce specific types of progeny. *Nat. Rev. Neurosci.* 15 615–627. 10.1038/nrn3767 25096185

[B22] Chen K. H. Boettiger A. N. Moffitt J. R. Wang S. Zhuang X. (2015). Spatially resolved, highly multiplexed RNA profiling in single cells. *Science* 348:aaa6090. 10.1126/science.aaa6090 25858977 PMC4662681

[B23] Cheung T. H. Rando T. A. (2013). Molecular regulation of stem cell quiescence. *Nat. Rev. Mol. Cell Biol.* 14 329–340. 10.1038/nrm3591.Molecular 23698583 PMC3808888

[B24] Cho I. J. Lui P. P. W. Obajdin J. Riccio F. Stroukov W. Willis T. L. (2019). Mechanisms, hallmarks, and implications of stem cell quiescence. *Stem Cell Rep.* 12 1190–1200. 10.1016/j.stemcr.2019.05.012 31189093 PMC6565921

[B25] Chow K.-H. K. Budde M. W. Granados A. A. Cabrera M. Yoon S. Cho S. (2021). Imaging cell lineage with a synthetic digital recording. *Science* 372:eabb3099. 10.1126/science.abb3099 33833095

[B26] Clavreul S. Abdeladim L. Hernández-Garzón E. Niculescu D. Durand J. Ieng S. H. (2019). Cortical astrocytes develop in a plastic manner at both clonal and cellular levels. *Nat. Commun.* 10 1–14. 10.1038/s41467-019-12791-5 31653848 PMC6814723

[B27] Clevers H. Rafelski S. Elowitz M. Lein E. (2017). What is your conceptual definition of “Cell Type” in the context of a mature organism? *Cell Syst.* 4 255–259. 10.1016/j.cels.2017.03.006 28334573

[B28] Cohen M. Briscoe J. Blassberg R. (2013). Morphogen interpretation: the transcriptional logic of neural tube patterning. *Curr. Opin. Genet. Dev.* 23 423–428. 10.1016/j.gde.2013.04.003 23725799

[B29] Collinet C. Lecuit T. (2021). Programmed and self-organized flow of information during morphogenesis. *Nat. Rev. Mol. Cell Biol.* 22 245–265. 10.1038/s41580-020-00318-6 33483696

[B30] Cotterell J. Vila-Cejudo M. Batlle-Morera L. Sharpe J. (2020). Endogenous CRISPR/Cas9 arrays for scalable whole-organism lineage tracing. *Development* 147:dev184481. 10.1242/dev.184481 32398353

[B31] Davidson E. H. Erwin D. H. (2006). Gene regulatory networks and the evolution of animal body plans. *Science* 311 796–801. 10.1126/science.1126454 16469913

[B32] Delile J. Rayon T. Melchionda M. Edwards A. Briscoe J. Sagner A. (2019). Single cell transcriptomics reveals spatial and temporal dynamics of gene expression in the developing mouse spinal cord. *Development* 146:dev173807. 10.1242/dev.173807 30846445 PMC6602353

[B33] Deneen B. Ho R. Lukaszewicz A. Hochstim C. J. Gronostajski R. M. Anderson D. J. (2006). The Transcription factor NFIA controls the onset of gliogenesis in the developing spinal cord. *Neuron* 52 953–968. 10.1016/j.neuron.2006.11.019 17178400

[B34] Desai A. R. Mcconnell S. K. (2000). Progressive restriction in fate potential by neural progenitors during cerebral cortical development. *Development* 127 2863–2872. 10851131 10.1242/dev.127.13.2863

[B35] Dessaud E. McMahon A. P. Briscoe J. (2008). Pattern formation in the vertebrate neural tube: a sonic hedgehog morphogen-regulated transcriptional network. *Development* 135 2489–2503. 10.1242/dev.009324 18621990

[B36] Dessaud E. Ribes V. Balaskas N. Yang L. L. Pierani A. Kicheva A. (2010). Dynamic assignment and maintenance of positional identity in the ventral neural tube by the morphogen sonic hedgehog. *PLoS Biol.* 8:e1000382. 10.1371/journal.pbio.1000382 20532235 PMC2879390

[B37] Dessaud E. Yang L. L. Hill K. Cox B. Ulloa F. Ribeiro A. (2007). Interpretation of the sonic hedgehog morphogen gradient by a temporal adaptation mechanism. *Nature* 450 717–720. 10.1038/nature06347 18046410

[B38] Dyballa S. Savy T. Germann P. Mikula K. Remesikova M. Špir R. (2017). Distribution of neurosensory progenitor pools during inner ear morphogenesis unveiled by cell lineage reconstruction. *eLife* 6:e22268. 10.7554/eLife.22268 28051766 PMC5243114

[B39] Enver T. Pera M. Peterson C. Andrews P. W. (2009). Stem cell states, fates, and the rules of attraction. *Cell Stem Cell* 4 387–397. 10.1016/j.stem.2009.04.011 19427289

[B40] Esain V. Postlethwait J. H. Charnay P. Ghislain J. (2010). FGF-receptor signalling controls neural cell diversity in the zebrafish hindbrain by regulating olig2 and sox9. *Development* 137 33–42. 10.1242/dev.038026 20023158 PMC2796930

[B41] Espinosa-Medina I. Garcia-Marques J. Cepko C. Lee T. (2019). High-throughput dense reconstruction of cell lineages. *Open Biol.* 9:190229. 10.1098/rsob.190229 31822210 PMC6936253

[B42] Fagan M. B. (2012). Waddington redux: models and explanation in stem cell and systems biology. *Biol. Philos.* 27 179–213. 10.1007/s10539-011-9294-y

[B43] Faure E. Savy T. Rizzi B. Melani C. Stašová O. Fabrèges D. (2016). A workflow to process 3D+time microscopy images of developing organisms and reconstruct their cell lineage. *Nat. Commun.* 7 1–10. 10.1038/ncomms9674 26912388 PMC4773431

[B44] Feng L. Hatten M. E. Heintz N. (1994). Brain lipid-binding protein (BLBP): a novel signaling system in the developing mammalian CNS. *Neuron* 12 895–908. 10.1016/0896-6273(94)90341-7 8161459

[B45] Figueres-Oñate M. García-Marqués J. López-Mascaraque L. (2016). UbC-StarTrack, a clonal method to target the entire progeny of individual progenitors. *Sci. Rep.* 6:33896. 10.1038/srep33896 27654510 PMC5031994

[B46] Figueres-Oñate M. Sánchez-González R. López-Mascaraque L. (2020). Deciphering neural heterogeneity through cell lineage tracing. *Cell. Mol. Life Sci.* 78 1971–1982. 10.1007/s00018-020-03689-3 33151389 PMC7966193

[B47] Fogarty M. Richardson W. D. Kessaris N. (2005). A subset of oligodendrocytes generated from radial glia in the dorsal spinal cord. *Development* 132 1951–1959. 10.1242/dev.01777 15790969

[B48] Franco S. J. Gil-Sanz C. Martinez-Garay I. Espinosa A. Harkins-Perry S. R. Ramos C. (2012). Fate-restricted neural progenitors in the mammalian cerebral cortex. *Science* 337 746–749. 10.1126/science.1223616 22879516 PMC4287277

[B49] Frantz G. D. McConnell S. K. (1996). Restrictions of late cerebral corticl progenitor cells to an upper-layer fate. *Neuron* 17 55–61. 10.1016/s0896-6273(00)80280-9 8755478

[B50] Frieda K. L. Linton J. M. Hormoz S. Choi J. Chow K. H. K. Singer Z. S. (2017). Synthetic recording and in situ readout of lineage information in single cells. *Nature* 541 107–111. 10.1038/nature20777 27869821 PMC6487260

[B51] Fuentealba L. C. Rompani S. B. Parraguez J. I. Obernier K. Romero R. Cepko C. L. (2015). Embryonic origin of postnatal neural stem cells. *Cell* 161 1644–1655. 10.1016/j.cell.2015.05.041 26091041 PMC4475276

[B52] Furutachi S. Miya H. Watanabe T. Kawai H. Yamasaki N. Harada Y. (2015). Slowly dividing neural progenitors are an embryonic origin of adult neural stem cells. *Nat. Neurosci.* 18 657–665. 10.1038/nn.3989 25821910

[B53] Gao P. Postiglione M. P. Krieger T. G. Hernandez L. Wang C. Han Z. (2014). Deterministic progenitor behavior and unitary production of neurons in the neocortex. *Cell* 159 775–788. 10.1016/j.cell.2014.10.027 25417155 PMC4225456

[B54] Garcia-Marques J. Espinosa-Medina I. Ku K. Y. Yang C. P. Koyama M. Yu H. H. (2020). A programmable sequence of reporters for lineage analysis. *Nat. Neurosci.* 23 1618–1628. 10.1038/s41593-020-0676-9 32719561

[B55] Garcia-Marques J. Espinosa-Medina I. Lee T. (2021). The art of lineage tracing: from worm to human. *Prog. Neurobiol.* 199:101966. 10.1016/j.pneurobio.2020.101966 33249090

[B56] García-Marqués J. López-Mascaraque L. (2013). Clonal identity determines astrocyte cortical heterogeneity. *Cereb. Cortex* 23 1463–1472. 10.1093/cercor/bhs134 22617854

[B57] Gensert J. M. Goldman J. E. (2001). Heterogeneity of cycling glial progenitors in the adult mammalian cortex and white matter. *J. Neurobiol.* 48 75–86. 10.1002/neu.1043 11438938

[B58] Gorski J. A. Talley T. Qiu M. Puelles L. Rubenstein J. L. R. Jones K. R. (2002). Cortical excitatory neurons and glia, but not GABAergic neurons, are produced in the Emx1-expressing lineage. *J. Neurosci.* 22 6309–6314. 10.1523/jneurosci.22-15-06309.2002 12151506 PMC6758181

[B59] Götz M. Huttner W. B. (2005). The cell biology of neurogenesis. *Nat. Rev. Mol. Cell Biol.* 6 777–788. 10.1038/nrm1739 16314867

[B60] Guerrero P. Perez-Carrasco R. Zagorski M. Page D. Kicheva A. Briscoe J. (2019). Neuronal differentiation influences progenitor arrangement in the vertebrate neuroepithelium. *Development* 146:dev176297. 10.1242/dev.176297 31784457 PMC6918779

[B61] Guillemot F. (2007). Spatial and temporal specification of neural fates by transcription factor codes. *Development* 134 3771–3780. 10.1242/dev.006379 17898002

[B62] Guo C. Eckler M. J. McKenna W. L. McKinsey G. L. Rubenstein J. L. R. Chen B. (2013). Fezf2 expression identifies a multipotent progenitor for neocortical projection neurons, astrocytes and oligodendrocytes. *Neuron* 80 1–7. 10.1016/j.neuron.2013.09.037.Fezf2 24314728 PMC3857588

[B63] Hansen A. H. Duellberg C. Mieck C. Loose M. Hippenmeyer S. (2017). Cell polarity in cerebral cortex development—cellular architecture shaped by biochemical networks. *Front. Cell. Neurosci.* 11:176. 10.3389/fncel.2017.00176 28701923 PMC5487411

[B64] Hartfuss E. Galli R. Heins N. Götz M. (2001). Characterization of CNS precursor subtypes and radial glia. *Dev. Biol.* 229 15–30. 10.1006/dbio.2000.9962 11133151

[B65] Haubensak W. Attardo A. Denk W. Huttner W. B. (2004). Neurons arise in the basal neuroepithelium of the early mammalian telencephalon: a major site of neurogenesis. *Proc. Natl. Acad. Sci. U.S.A.* 101 3196–3201. 10.1073/pnas.0308600100 14963232 PMC365766

[B66] He J. Zhang G. Almeida A. D. Cayouette M. Simons B. D. Harris W. A. (2012). How variable clones build an invariant retina. *Neuron* 75 786–798. 10.1016/j.neuron.2012.06.033 22958820 PMC3485567

[B67] He W. Ingraham C. Rising L. Goderie S. Temple S. (2001). Multipotent stem cells from the mouse basal forebrain contribute GABAergic neurons and oligodendrocytes to the cerebral cortex during embryogenesis. *J. Neurosci.* 21 8854–8862. 10.1523/jneurosci.21-22-08854.2001 11698597 PMC6762260

[B68] Hochstim C. Deneen B. Lukaszewicz A. Zhou Q. David J. (2008). The spinal cord contains positionally distinct astrocyte subtypes whose identities are specified by a homeodomain transcriptional code. *Cell* 133 510–522. 10.1016/j.cell.2008.02.046 18455991 PMC2394859

[B69] Huttner W. B. Kosodo Y. (2005). Symmetric versus asymmetric cell division during neurogenesis in the developing vertebrate central nervous system. *Curr. Opin. Cell Biol.* 17 648–657. 10.1016/j.ceb.2005.10.005 16243506

[B70] Jessell T. M. (2000). Neuronal specification in the spinal cord: inductive signals and transcriptional codes. *Nat. Rev. Genet.* 1 20–29. 10.1038/35049541 11262869

[B71] Kang P. Lee H. K. Glasgow S. M. Finley M. Donti T. Gaber Z. B. (2012). Sox9 and NFIA coordinate a transcriptional regulatory cascade during the initiation of gliogenesis. *Neuron* 74 79–94. 10.1016/j.neuron.2012.01.024.Sox9 22500632 PMC3543821

[B72] Keller P. J. Ahrens M. B. (2015). Visualizing whole-brain activity and development at the single-cell level using light-sheet microscopy. *Neuron* 85 462–483. 10.1016/j.neuron.2014.12.039 25654253

[B73] Keller P. J. Schmidt A. D. Wittbrodt J. Stelzer E. H. K. (2008). Reconstruction of zebrafish early embryonic development by scanned light sheet microscopy. *Science* 322 1065–1069. 10.1126/science.1162493 18845710

[B74] Kessaris N. Fogarty M. Iannarelli P. Grist M. Wegner M. Richardson W. D. (2006). Competing waves of oligodendrocytes in the forebrain and postnatal elimination of an embryonic lineage. *Nat. Neurosci.* 9 173–179. 10.1038/nn1620 16388308 PMC6328015

[B75] Kessaris N. Pringle N. Richardson W. D. (2008). Specification of CNS glia from neural stem cells in the embryonic neuroepithelium. *Philos. Trans. R. Soc. B Biol. Sci.* 363 71–85. 10.1098/rstb.2006.2013 17282992 PMC2605487

[B76] Kicheva A. Bollenbach T. Ribeiro A. Valle H. P. Lovell-R. (2014). Coordination of progenitor specification and growth in the mouse and chick spinal cord. *Sciecne* 345 1–24. 10.1126/science.1254927.Coordination PMC4228193 25258086

[B77] Kicheva A. Briscoe J. (2015). Developmental pattern formation in phases. *Trends Cell Biol.* 25 579–591. 10.1016/j.tcb.2015.07.006 26410404

[B78] Kiecker C. Lumsden A. (2005). Compartments and their boundaries in vertebrate brain development. *Nat. Rev. Neurosci.* 6 553–564. 10.1038/nrn1702 15959467

[B79] Klingler E. Jabaudon D. (2020). Do progenitors play dice? *eLife* 8:e51381. 10.7554/eLife.51381 31951199 PMC6968926

[B80] Kohwi M. Doe C. Q. (2013). Temporal fate specification and neural progenitor competence during development. *Nat. Rev. Neurosci.* 14 823–838. 10.1038/nrn3618 24400340 PMC3951856

[B81] Kutejova E. Sasai N. Shah A. Gouti M. Briscoe J. (2016). Neural progenitors adopt specific identities by directly repressing all alternative progenitor transcriptional programs. *Dev. Cell* 36 639–653. 10.1016/j.devcel.2016.02.013 26972603 PMC4819439

[B82] Lanjakornsiripan D. Pior B. J. Kawaguchi D. Furutachi S. Tahara T. Katsuyama Y. (2018). Layer-specific morphological and molecular differences in neocortical astrocytes and their dependence on neuronal layers. *Nat. Commun.* 9:1623. 10.1038/s41467-018-03940-3 29691400 PMC5915416

[B83] Laurenti E. Göttgens B. (2018). From haematopoietic stem cells to complex differentiation landscapes. *Nature* 553 418–426. 10.1038/nature25022 29364285 PMC6555401

[B84] Le Dréau G. Martí E. (2013). The multiple activities of BMPs during spinal cord development. *Cell. Mol. Life Sci.* 70 4293–4305. 10.1007/s00018-013-1354-9 23673983 PMC11113619

[B85] Le Dréau G. Saade M. Gutiérrez-Vallejo I. Martí E. (2014). The strength of SMAD1/5 activity determines the mode of stem cell division in the developing spinal cord. *J. Cell Biol.* 204 591–605. 10.1083/jcb.201307031 24515346 PMC3926951

[B86] Livet J. Weissman T. A. Kang H. Draft R. W. Lu J. Bennis R. A. (2007). Transgenic strategies for combinatorial expression of fluorescent proteins in the nervous system. *Nature* 450 56–62. 10.1038/nature06293 17972876

[B87] Llorca A. Ciceri G. Beattie R. Wong F. K. Diana G. Serafeimidou-Pouliou E. (2019). A stochastic framework of neurogenesis underlies the assembly of neocortical cytoarchitecture. *eLife* 8:51381. 10.7554/eLife.51381 PMC6968929 31736464

[B88] Loulier K. Barry R. Mahou P. Franc Y. L. Supatto W. Matho K. S. (2014). multiplex cell and lineage tracking with combinatorial labels. *Neuron* 81 505–520. 10.1016/j.neuron.2013.12.016 24507188

[B89] Lu D. C. Niu T. Alaynick W. A. (2015). Molecular and cellular development of spinal cord locomotor circuitry. *Front. Mol. Neurosci.* 8:25. 10.3389/fnmol.2015.00025 26136656 PMC4468382

[B90] Luengo-Oroz M. A. Ledesma-Carbayo M. J. Peyriéras N. Santos A. (2011). Image analysis for understanding embryo development: a bridge from microscopy to biological insights. *Curr. Opin. Genet. Dev.* 21 630–637. 10.1016/j.gde.2011.08.001 21893410

[B91] Malatesta P. Götz M. (2013). Radial glia - from boring cables to stem cell stars. *Development* 140 483–486. 10.1242/dev.085852 23293279

[B92] McArthur K. L. Fetcho J. R. (2017). Key features of structural and functional organization of *Zebrafish* facial motor neurons are resilient to disruption of neuronal migration. *Curr. Biol.* 27 1746–1756. 10.1016/j.cub.2017.05.033 28602649 PMC5541779

[B93] McConnell S. K. Kaznowski C. E. (1991). Cell cycle dependence of laminar determination in developing neocortex. *Science* 254 282–285. 10.1126/science.1925583 1925583

[B94] McKenna A. Findlay G. M. Gagnon J. A. Horwitz M. S. Schier A. F. Shendure J. (2016). Whole-organism lineage tracing bycombinatorial and cumulativegenome editing. *Science* 353 1–13. 10.1126/science.aaf7907 PMC4967023 27229144

[B95] Meijer D. H. Kane M. F. Mehta S. Liu H. Harrington E. Taylor C. M. (2012). Separated at birth? the functional and molecular divergence of OLIG1 and OLIG2. *Nat. Rev. Neurosci.* 13 819–831. 10.1038/nrn3386 23165259 PMC3733228

[B96] Mihalas A. B. Hevner R. F. (2018). Clonal analysis reveals laminar fate multipotency and daughter cell apoptosis of mouse cortical intermediate progenitors. *Development* 145:e164335. 10.1242/dev.164335 30217810 PMC6141770

[B97] Miyata T. Kawaguchi A. Okano H. Ogawa M. (2001). Asymmetric inheritance of radial glial fibers by cortical neurons. *Neuron* 31 727–741. 10.1016/S0896-6273(01)00420-2 11567613

[B98] Mo Z. Zecevic N. (2009). Human fetal radial glia cells generate oligodendrocytes in vitro. *Glia* 57 490–498. 10.1002/glia.20775 18814269 PMC2644732

[B99] Noctor S. C. Martinez-Cerdeño V. Ivic L. Kriegstein A. R. (2004). Cortical neurons arise in symmetric and asymmetric division zones and migrate through specific phases. *Nat. Neurosci.* 7 136–144. 10.1038/nn1172 14703572

[B100] Olivier N. Luengo-Oroz M. A. Duloquin L. Faure E. Savy T. Veilleux I. (2010). Cell lineage reconstruction of early *Zebrafish embryos* using label-free nonlinear microscopy. *Science* 329 967–971. 10.1126/science.1189428 20724640

[B101] Oosterveen T. Kurdija S. Ensterö M. Uhde C. W. Bergsland M. Sandberg M. (2013). SoxB1-driven transcriptional network underlies neural-specific interpretation of morphogen signals. *Proc. Natl. Acad. Sci. U.S.A.* 110 7330–7335. 10.1073/pnas.1220010110 23589857 PMC3645538

[B102] Penisson M. Ladewig J. Belvindrah R. Francis F. (2019). Genes and mechanisms involved in the generation and amplification of basal radial glial cells. *Front. Cell. Neurosci.* 13:381. 10.3389/fncel.2019.00381 31481878 PMC6710321

[B103] Peterson K. A. Nishi Y. Ma W. Vedenko A. Shokri L. Zhang X. (2012). Neural-specific Sox2 input and differential Gli-binding affinity provide context and positional information in Shh-directed neural patterning. *Genes Dev.* 26 2802–2816. 10.1101/gad.207142.112 23249739 PMC3533082

[B104] Pujala A. Koyama M. (2019). Chronology-based architecture of descending circuits that underlie the development of locomotor repertoire after birth. *eLife* 8:e42135. 10.7554/eLife.42135 30801247 PMC6449084

[B105] Raj B. Wagner D. E. McKenna A. Pandey S. Klein A. M. Shendure J. (2018). Simultaneous single-cell profiling of lineages and cell types in the vertebrate brain. *Nat. Biotechnol.* 36 442–450. 10.1038/nbt.4103 29608178 PMC5938111

[B106] Rakic P. (1974). Neurons in rhesus monkey visual cortex: sustematic relation between time of origin and eventual disposition. *Science* 183 425–427. 10.1126/science.183.4123.425 4203022

[B107] Rakic P. (2009). Evolution of the neocortex: a perspective from developmental biology. *Nat. Rev. Neurosci.* 10 724–735. 10.1038/nrn2719 19763105 PMC2913577

[B108] Rayon T. Briscoe J. (2021). Cross-species comparisons and in vitro models to study tempo in development and homeostasis. *Interface Focus* 11:20200069. 10.1098/rsfs.2020.0069 34055305 PMC8086913

[B109] Rayon T. Stamataki D. Perez-Carrasco R. Garcia-Perez L. Barrington C. Melchionda M. (2020). Species-specific pace of development is associated with differences in protein stability. *Science* 369:eaba7667. 10.1126/science.aba7667 32943498 PMC7116327

[B110] Ribes V. Briscoe J. (2009). Establishing and interpreting graded Sonic Hedgehog signaling during vertebrate neural tube patterning: the role of negative feedback. *Cold Spring Harb. Perspect. Biol.* 1 1–16. 10.1101/cshperspect.a002014 20066087 PMC2742090

[B111] Robertis E. M. D. (2006). Spemann’s organizer and self- regulation in amphibian embryos. *Nat. Rev. Mol. Cell Biol.* 7 296–302. 10.1038/nrm1855 16482093 PMC2464568

[B112] Rogers K. W. Schier A. F. (2011). Morphogen gradients: from generation to interpretation. *Annu. Rev. Cell Dev. Biol.* 27 377–407. 10.1146/annurev-cellbio-092910-154148 21801015

[B113] Rulands S. Iglesias-Gonzalez A. B. Boije H. (2018). Deterministic fate assignment of *Müller glia* cells in the *Zebrafish retina* suggests a clonal backbone during development. *Eur. J. Neurosci.* 48 3597–3605. 10.1111/ejn.14257 30408243 PMC6588021

[B114] Saade M. Gonzalez-Gobartt E. Escalona R. Usieto S. Martí E. (2017). Shh-mediated centrosomal recruitment of PKA promotes symmetric proliferative neuroepithelial cell division. *Nat. Cell Biol.* 19 493–503. 10.1038/ncb3512 28446817

[B115] Saade M. Gutiérrez-Vallejo I. LeDréau G. Rabadán M. A. Miguez D. G. Buceta J. (2013). Sonic hedgehog signaling switches the mode of division in the developing nervous system. *Cell Rep.* 4 492–503. 10.1016/j.celrep.2013.06.038 23891002

[B116] Sagner A. Briscoe J. (2019). Establishing neuronal diversity in the spinal cord: a time and a place. *Development* 146:e182154. 10.1242/dev.182154 31767567

[B117] Slack J. M. W. (2002). Conrad hal waddington: the last renaissance biologist? *Nat. Rev. Genet.* 3 889–895. 10.1038/nrg933 12415319

[B118] Snippert H. J. van der Flier L. G. Sato T. van Es J. H. van den Born M. Kroon-Veenboer C. (2010). Intestinal crypt homeostasis results from neutral competition between symmetrically dividing Lgr5 stem cells. *Cell* 143 134–144. 10.1016/j.cell.2010.09.016 20887898

[B119] Soula C. Danesin C. Kan P. Grob M. Poncet C. Cochard P. (2001). Distinct sites of origin of oligodendrocytes and somatic motoneurons in the chick spinal cord: oligodendrocytes arise from Nkx2.2-expressing progenitors by a Shh-dependent mechanism. *Development* 128 1369–1379. 10.1242/dev.128.8.1369 11262237

[B120] Spanjaard B. Hu B. Mitic N. Olivares-Chauvet P. Janjuha S. Ninov N. (2018). Simultaneous lineage tracing and cell-type identification using CrIsPr-Cas9-induced genetic scars. *Nat. Biotechnol.* 36 469–473. 10.1038/nbt.4124 29644996 PMC5942543

[B121] Spassky N. Goujet-Zalc C. Parmantier E. Olivier C. Martinez S. Ivanova A. (1998). Multiple restricted origin of oligodendrocytes. *J. Neurosci.* 18 8331–8343. 10.1523/jneurosci.18-20-08331.1998 9763477 PMC6792828

[B122] Subramanian L. Bershteyn M. Paredes M. F. Kriegstein A. R. (2017). Dynamic behaviour of human neuroepithelial cells in the developing forebrain. *Nat. Commun.* 8:14167. 10.1038/ncomms14167 28139695 PMC5290330

[B123] Sueda R. Imayoshi I. Harima Y. Kageyama R. (2019). High Hes1 expression and resultant Ascl1 suppression regulate quiescent vs. active neural stem cells in the adult mouse brain. *Genes Dev.* 33 511–523. 10.1101/gad.323196.118 30862661 PMC6499325

[B124] Sulston J. E. Schierenberg E. White J. G. Thomson J. N. (1983). The embryonic cell lineage of the nematode *Caenorhabditis elegans*. *Dev. Biol.* 100 64–119. 10.1016/0012-1606(83)90201-4 6684600

[B125] Takahashi K. Yamanaka S. (2016). A decade of transcription factor-mediated reprogramming to pluripotency. *Nat. Rev. Mol. Cell Biol.* 17 183–193. 10.1038/nrm.2016.8 26883003

[B126] Takahashi T. Goto T. Miyama S. Nowakowski R. S. Caviness V. S. (1999). Sequence of neuron origin and neocortical laminar fate: relation to cell cycle of origin in the developing murine cerebral wall. *J. Neurosci.* 19 10357–10371. 10.1523/jneurosci.19-23-10357.1999 10575033 PMC6782435

[B127] Tambalo M. Mitter R. Wilkinson D. G. (2020). A single cell transcriptome atlas of the developing zebrafish hindbrain. *Development* 147:e184143. 10.1242/dev.184143 32094115 PMC7097387

[B128] Tan S. S. Kalloniatis M. Truong H. T. Binder M. D. Cate H. S. Kilpatrick T. J. (2009). Oligodendrocyte positioning in cerebral cortex is independent of projection neuron layering. *Glia* 57 1024–1030. 10.1002/glia.20826 19062175

[B129] Tarabykin V. Stoykova A. Usman N. Gruss P. (2001). Cortical upper layer neurons derive from the subventricular zone as indicated by Svet1 gene expression. *Development* 128 1983–1993. 10.1242/dev.128.11.1983 11493521

[B130] Tassy O. Daian F. Hudson C. Bertrand V. Lemaire P. (2006). A quantitative approach to the study of cell shapes and interactions during early chordate embryogenesis. *Curr. Biol.* 16 345–358. 10.1016/j.cub.2005.12.044 16488868

[B131] Taverna E. Götz M. Huttner W. B. (2014). The cell biology of neurogenesis: toward an understanding of the development and evolution of the neocortex. *Annu. Rev. Cell Dev. Biol.* 30 465–502. 10.1146/annurev-cellbio-101011-155801 25000993

[B132] Tekki-Kessaris N. Woodruff R. Hall A. C. Gaffield W. Kimura S. Stiles C. D. (2001). Hedgehog-dependent oligodendrocyte lineage specification in the telencephalon. *Development* 128 2545–2554. 10.1242/dev.128.13.2545 11493571

[B133] Telley L. Agirman G. Prados J. Amberg N. Fièvre S. Oberst P. (2019). Temporal patterning of apical progenitors and their daughter neurons in the developing neocortex. *Science* 364:eaav2522. 10.1126/science.aav2522 31073041

[B134] Than-Trong E. Bally-Cuif L. (2015). Radial glia and neural progenitors in the adult zebrafish central nervous system. *Glia* 63 1406–1428. 10.1002/glia.22856 25976648

[B135] Than-Trong E. Kiani B. Dray N. Ortica S. Simons B. Rulands S. (2020). Lineage hierarchies and stochasticity ensure the long-term maintenance of adult neural stem cells. *Sci. Adv.* 6 26–29. 10.1126/sciadv.aaz5424 32426477 PMC7190328

[B136] Trapnell C. (2015). Defining cell types and states with single-cell genomics. *Genome Res.* 25 1491–1498. 10.1101/gr.190595.115 26430159 PMC4579334

[B137] Ulloa F. Briscoe J. (2007). Morphogens and the control of cell proliferation and patterning in the spinal cord. *Cell Cycle* 6 2640–2649. 10.4161/cc.6.21.4822 17912034

[B138] Urbán N. Cheung T. H. (2021). Stem cell quiescence: the challenging path to activation. *Development* 148:dev165084. 10.1242/dev.165084 33558315 PMC7888710

[B139] van Velthoven C. T. J. Rando T. A. (2019). Stem cell quiescence: dynamism, restraint, and cellular idling. *Cell Stem Cell* 24 213–225. 10.1016/j.stem.2019.01.001 30735649 PMC6413865

[B140] Waddington C. H. (1957). *The Strategy of the Genes: A Discussion of Some Aspects of Theoretical Biology. George.* London: Allen and Unwin.

[B141] Wan Y. Wei Z. Looger L. L. Koyama M. Druckmann S. Keller P. J. (2019). Single-cell reconstruction of emerging population activity in an entire developing circuit. *Cell* 179 355–372. 10.1016/j.cell.2019.08.039 31564455 PMC7055533

[B142] Wang X. Allen W. E. Wright M. A. Sylwestrak E. L. Samusik N. Vesuna S. (2018). Three-dimensional intact-tissue sequencing of single-cell transcriptional states. *Science* 361:eaat5691. 10.1126/science.aat5691 29930089 PMC6339868

[B143] Weissman T. A. Sanes J. R. Lichtman J. W. Livet J. (2011). Generating and imaging multicolor brainbow mice. *Cold Spring Harb. Protoc.* 6 763–769. 10.1101/pdb.top114 21724826

[B144] Wolff C. Tinevez J. Y. Pietzsch T. Stamataki E. Harich B. Guignard L. (2018). Multi-view light-sheet imaging and tracking with the MaMuT software reveals the cell lineage of a direct developing arthropod limb. *eLife* 7:34410. 10.7554/eLife.34410 29595475 PMC5929908

[B145] Yu H. H. Chen C. H. Shi L. Huang Y. Lee T. (2009). Twin-spot MARCM to reveal the developmental origin and identity of neurons. *Nat. Neurosci.* 12 947–953. 10.1038/nn.2345 19525942 PMC2701974

[B146] Zannino D. A. Appel B. (2009). Olig2 + precursors produce abducens motor neurons and oligodendrocytes in the zebrafish hindbrain. *J. Neurosci.* 29 2322–2333. 10.1523/JNEUROSCI.3755-08.2009 19244509 PMC2720165

[B147] Zechner C. Nerli E. Norden C. (2020). Stochasticity and determinism in cell fate decisions. *Development* 147 1–8. 10.1242/dev.181495 32669276

[B148] Zhang M. Eichhorn S. W. Zingg B. Yao Z. Zeng H. Hongwei D. (2020). Molecular, spatial and projection diversity of neurons in primary motor cortex revealed by in situ single-cell transcriptomics. *bioRxiv* [Preprint]. 10.1101/2020.06.04.105700

[B149] Zhang X. Mennicke C. V. Xiao G. Beattie R. Haider M. A. Hippenmeyer S. (2020). Clonal analysis of gliogenesis in the cerebral cortex reveals stochastic expansion of glia and cell autonomous responses to egfr dosage. *Cells* 9:2662. 10.3390/cells9122662 33322301 PMC7764668

[B150] Zong H. Espinosa J. S. Su H. H. Muzumdar M. D. Luo L. (2005). Mosaic analysis with double markers in mice. *Cell* 121 479–492. 10.1016/j.cell.2005.02.012 15882628

